# Personal

**Published:** 1897-03

**Authors:** 


### Personal.

Dr. Henry P. Frost has been appointed assistant physician at the Buffalo State Hospital, vice Dr. Percy Bryant, appointed superintendent of the Manhattan State Hospital, New York City. This appointment was made under the civil service law, which confers authority on the superintendent of the State Hospital to appoint his assistants from those already in service.

Dr. Frost comes to this new position from the Willard State Hospital and has been six years in service. He is a Virginian by birth, but has lived in New York a number of years.

Dr. Bryant was a most competent official, but Dr. Hurd seems to have been fortunate in obtaining so worthy a successor to his accomplished first assistant, whose many Buffalo friends part from him with regret.

